# Realizing drug repositioning by adapting a recommendation system to handle the process

**DOI:** 10.1186/s12859-018-2142-1

**Published:** 2018-04-12

**Authors:** Makbule Guclin Ozsoy, Tansel Özyer, Faruk Polat, Reda Alhajj

**Affiliations:** 1 Department of Computer Engineering, Middle East Technical University, Ankara, Turkey; 2 Department of Computer Engineering, TOBB University, Ankara, Turkey; 3 Department of Computer Science, University of Calgary, Calgary, AB Canada

**Keywords:** Drug repositioning, Multiple data sources, Multiple features, Pareto dominance, Collaborative filtering, Recommendation systems

## Abstract

**Background:**

Drug repositioning is the process of identifying new targets for known drugs. It can be used to overcome problems associated with traditional drug discovery by adapting existing drugs to treat new discovered diseases. Thus, it may reduce associated risk, cost and time required to identify and verify new drugs. Nowadays, drug repositioning has received more attention from industry and academia. To tackle this problem, researchers have applied many different computational methods and have used various features of drugs and diseases.

**Results:**

In this study, we contribute to the ongoing research efforts by combining multiple features, namely chemical structures, protein interactions and side-effects to predict new indications of target drugs. To achieve our target, we realize drug repositioning as a recommendation process and this leads to a new perspective in tackling the problem. The utilized recommendation method is based on Pareto dominance and collaborative filtering. It can also integrate multiple data-sources and multiple features. For the computation part, we applied several settings and we compared their performance. Evaluation results show that the proposed method can achieve more concentrated predictions with high precision, where nearly half of the predictions are true.

**Conclusions:**

Compared to other state of the art methods described in the literature, the proposed method is better at making right predictions by having higher precision. The reported results demonstrate the applicability and effectiveness of recommendation methods for drug repositioning.

## Background

Traditional drug discovery approaches are characterized by high cost and high risk [22]. In 2010, some researchers, e.g., [9], stated that bringing a new drug to the market takes about 15 years and costs between $800 million to $1 billion. A recent study, published in 2014 [7], revealed that developing a new medicine and getting its market approval takes more than 10 years and costs more than $2.5 billion. In response to these costs, drug repositioning has recently received considerable attention as a good alternative which could reduce both time and cost associated with seeking new drugs for emerging diseases. Instead, existing drugs may be adapted as less risky alternatives.

Drug repositioning can be defined as the process of identifying new targets for known drugs [22]. It does not aim to replace traditional drug discovery research, but aims to complement them ([31, 35]). Researchers stated in [9] that time required to develop a new drug can be reduced by 30–60% by adapting drug repositioning. Having knowledge of unknown but more probable drug-disease relations may help researchers in drug industry to conduct more targeted laboratory experiments and find out new targets for known drugs. Another advantage of drug repositioning compared to new drug development is that drug repositioning reduces risk because it deals with drugs which have already passed toxicity and other tests, and hence have been approved [37]. Some example drug repositioning cases are presented in [9]. For instance, Minoxidil was originally tested for hypertension and then was found useful for hair loss, Viagra was originally tested for angina and then was found useful for erectile dysfunction and pulmonary hypertension, Avastin was originally developed for metastatic colon cancer and non-small-cell lung cancer and then it was found useful for metastatic breast cancer. As a result of the above-mentioned advantages, drug repositioning has received more attention from industry and academia [9].

Nowadays, with the advancement in technology, researchers are more capable of reaching different types of biological data and complex networks which are composed of different types of interactions among biological components [10]. Using these data sources, many different computational methodologies may be used to predict possible new use-cases (repositions) for drugs. As described in the literature, most researchers tackled the problem by applying methods from data mining and machine learning. These methods use a single feature or combination of features to model drugs. Some example features used in the process are chemical structures of drugs, protein targets, side-effect profiles and gene expression profiles [41].

In this study, we adapted a method from the recommendation systems literature to handle the drug repositioning problem. The utilized method has already been applied to produce successful recommendation systems in various domains, including location recommendation [29] and bioinformatics for predicting the structure of gene regulatory networks (GRNs) [30]. The recommendation method employed in this study is based on Pareto dominance and collaborative filtering. It is also capable of integrating multiple data-sources and multiple features. Inspiring from a state-of-the-art method for drug repositioning [41], we used three types of information; namely chemical properties, protein targets and side-effect profiles. For the calculations, we applied several different settings and we compared their performance results. The conducted experiments revealed some promising results which demonstrate the applicability and effectiveness of the proposed approach.

As described in the literature, identifying new targets for known drugs, namely drug repositioning, has recently received more attention from industry and academia. The work described in [9] classifies computational drug repositioning methods into two categories: namely drug-based and disease-based approaches. Drug-based repositioning methods initiate their analysis from chemical or pharmaceutical features of drugs. Disease-based repositioning methods initiate the analysis from symptomatology or pathology features of diseases. Drug repositioning methods use various features for the computations [41], e.g., Chemical structure of drugs, proteins and targets interaction networks, side-effect of drugs, gene expression levels and textual features.

There are many drug repositioning methods described in the literature. However, they mostly use only one feature: structural and chemical properties of a drug in relation to diseases it affects. Drugs with high chemical similarity can be used for drug repositioning [9]. The works described in [19, 27] are example methods that use chemical similarity for drug repositioning. Authors of the work described in [5] stated that common segments in protein-protein interaction and protein-targets interaction networks can reveal cross-reactions and can be used for drug repositioning. The works described in [20, 23] use protein-targets interaction networks. Side effects form physiological consequences of drugs’ biological activity; they can provide information on underlying pathways or physiological systems to which drugs are related [9]. Side-effect similarity between drugs may indicate physiological relatedness between them. The works described in [1, 40] use side-effect similarity of drugs for drug repositioning. Similarities at molecular level can also be used for drug repositioning [9]. For this purpose, the works described in [12, 13, 34] use gene expressions and molecular activity signatures. Some of the works described in the literature rely on text mining tools to connect drugs and diseases [32]. One such method is described in [2]. It applies text mining methods to associate query and matching terms related to diseases, genes, drugs, mutations and metabolites. It also ranks related sentences and abstracts.

Recent drug-repositioning methods combined multiple features to achieve better performance. For instance, the work described in [22] combined chemical and molecular features to find out similar drugs. The authors applied a bipartite graph based method to predict novel indications of drugs. Luo et al. [26] used drug-drug and disease-disease similarities to create a graph. Then they employed random-walk on this graph to extract new drug-disease relations. Lim et al. [24] used chemical and protein similarities to create a network of drug-disease relations. Then they used matrix factorization to decide on drugs which can be repurposed. They showed that their proposed method is highly scalable. Gottlieb et al. [11] used chemical structures, side effects and drug targets to calculate pairwise similarity of drugs. They used the calculated similarities as input features for a machine learning method, namely logistic regression. They predicted new drug-disease relations. Zhang et al. [41] used chemical, biological and phenotypical features to calculate drug-drug similarities which are used to find out k-nearest-neighbors. Then known targets of neighbors are used for drug repositioning. Qabaja et al. [32] combined information collected from gene expression profiling and text mining. They applied logistic regression to predict associations between drugs and diseases. Ozgur et al. [28] used text mining techniques to create a parse tree which was then used to create a protein-protein network. They also applied some social network analysis techniques (e.g., degree centrality, closeness) to prioritize genes’ effect on diseases. Rastegar-Mojarad et al. [33] also used text mining techniques to repurpose drugs. They collected user comments on drugs and diseases from social media; they applied a combination of machine learning and rule based approaches to extract candidates for drug repurposing. Recent research on big-data in bioinformatics can also reveal new ways to find new indications of known drugs. The work described in [15, 16] proposed new methods to identify damages and DNA breaks which are important for disease investigations and drug design. The work described in [18] focused on cancer disease and applied several different machine learning methods for data reduction and coding area selection, which is considered as key area for discovering the desired medicine. The research described in [14, 17] can be used for extracting drug-disease relations, which aim to predict the primary, secondary and tertiary protein structure and to handle large volume biological datasets.

Compared to the works described in the literature, in this paper we investigate the problem of drug repositioning from a different perspective which enriches the current literature related to this field and additionally confirms the results reported. In particular, we realize drug repositioning as a recommendation process. In other words, we argue that it is possible to recommend existing drugs for treating emerging diseases based on characteristics of new diseases as compared to characteristics of existing diseases in relationship with associated effective drugs. Thus, we apply a method from recommendation systems domain to tackle the drug repositioning problem. The employed method is able to integrate multiple data-sources and multiple features. Similar to the work of Zhang et al. [41], the proposed method first identifies drugs most similar to the target drug. Then, it uses known relations of neighbor drugs to predict new indications of the target drug. Unlike the work of Zhang et al. [41], we use a Pareto dominance and collaborative filtering based method, which has been already used as part of adapting recommendation systems to other domains, like venue recommendation and in bio-informatics to predict the structure of gene regulatory networks. Also, we have applied several settings for the calculation and we have compared the performance of the two methods.

The rest of this paper is organized as follows: “Methods” section, presents the proposed drug repositioning method. “Results and discussion” section, includes the evaluation process and the results. “Conclusions” section is conclusions and future work.

## Methods

The aim of this work is to predict new uses of known drugs by analyzing multiple features and multiple data sources. For this purpose, we adapted a recommendation system based method which has been successfully applied in other domains. Fortunately, the results reported from this study clearly demonstrate the effectiveness and applicability of recommendation methods for drug repositioning. In other words, the process could be easily mapped to recommending an existing drug for handling a new disease by studying characteristics of new diseases in link to already known diseases and their associated drugs. Zhang et al. [41] stated that similar drugs are indicators for similar diseases. Accordingly, in their work they inspired from similar diseases to reposition target drugs. Realizing the fact that this approach is similar to collaborative filtering in the recommendation systems domain, we adapted for drug repositioning a method that we previously proposed for classical recommendation purposes [29]. In the following subsections, we first present the proposed method in general, and then we describe steps of the method in details.

### Pareto dominance and collaborative filtering based prediction

The utilized recommendation method uses Pareto dominance and collaborative filtering approaches to predict future venue preferences (i.e, check-in locations) of target users. Its idea is based on the observation that similar users tend to visit similar venues. Accordingly, it would be acceptable to recommend to a target user venues that have been visited by similar users. As described in [30], we applied the same concept in the bioinformatics domain for predicting structure of gene regulatory networks. In the latter work, target genes are used instead of target users and accordingly regulated genes are predicted. The achieved results confirmed promising aspects of adapting a recommendation system to discover gene regulations.

**Fig. 1:**
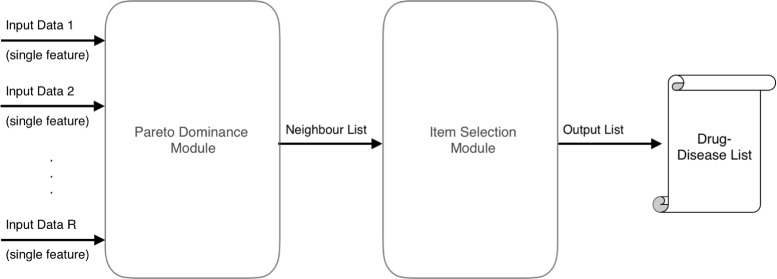
Design of the proposed method

The success achieved in studying gene regulatory networks motivated us to investigate the applicability of recommendation systems for drug repositioning. The overall design of the proposed method for drug repositioning is shown in Fig. 1, where the modules and their interactions are presented. The proposed method is composed of three main steps, namely similarity calculation, neighbor selection and item (disease) selection. In the similarity calculation step, each feature is used to determine similarity between drugs. Then, similarities are used to find most similar drugs, namely neighbors, by a Pareto dominance based method. Then known connections among neighbor drugs and indicated diseases are used for prediction. Reported at the end is a prediction list of target drugs and predicted diseases which could be treated by target drugs.

### Details of the proposed method

For the calculations performed in the process, we used three main features: namely chemical properties of drugs, protein targets, and side-effect profiles. In this section, we explain details of the various steps of the proposed method and how the above-mentioned features are used.

#### Similarity calculation

In this step, similarity between drugs is calculated for each type of features. We used several similarity measures in the calculation, namely Cosine similarity, Jaccard similarity and a similarity score based on Smith-Waterman sequence alignment. In this section, we present how these similarity measures are calculated. In the evaluation section, we present how these similarity measures have been used and combined, as well as their corresponding performance results.

Cosine similarity is calculated as depicted in Eq. 1, where *A* and *B* denote drugs. Drugs may be represented as vectors, where a vector contains one value per feature to reflect how a drug is related to the specific feature. Subscript *j* in Eq. 1 refers to individual values of a feature vector. For instance, for the “chemical properties” feature, a drug may be represented as a binary vector where values represent the existence/non-existence of a chemical structure. Similarity between two drugs can be calculated based on common chemical structures and the length of the feature vector.

1 $$ sim(A,B)=\frac{\sum\limits_{j=1}^{n} A_{j} \times B_{j}}{\sqrt{\sum\limits_{j=1}^{n} A_{j}^{2}} \times \sqrt{ \sum\limits_{j=1}^{n} B_{j}^{2}}}  $$

Jaccard similarity is calculated by invoking Eq. 2, where |*A*| represents length of the drug feature vector and |*AB*| represents size of common elements in the feature vector. This similarity measure is also called Tanimoto index/similarity when the feature vector is binary.

2 $$ sim(A,B)=\frac{|AB|}{|A| + |B| - |AB| }  $$

In the work of Zhang et al. [41], a similarity score based on Smith-Waterman sequence alignment is used. In this study, we also applied the same similarity measure when possible. As explained previously, drugs may be represented as a feature vector. Entries/elements of a vector themselves can be represented as sequences. For instance, a drug can be represented as a vector of proteins. Proteins themselves may be represented as a sequence of smaller biological elements. Similarity of these sequences, e.g., protein sequences, can be calculated by Smith-Waterman sequence alignment method. After having Smith-Waterman sequence alignment score, similarity among drugs can be calculated by the formula given in Eq. 3,^1^.

In Eq. 3, *V*(*A*) represents the feature vector of drug *A*, and each vector element is composed of a sequence of smaller elements, where these elements are represented as *V*_*i*_(*A*). Smith-Waterman sequence alignment score computed by Eq. 3 is denoted *sim*_*S*_*W*(*V*_*i*_(*A*),*V*_*j*_(*B*)).

3 $$ sim(A,B)=\frac{\sum\limits_{i=1}^{|V(A)|} \sum\limits_{j=1}^{|V(B)|} sim_{S}W(V_{i}(A),V_{j}(B))} {|V(A)| \times |V(B)|}  $$

#### Neighbor selection

In this step, drugs most similar to the target drug (i.e., its neighbors) are selected. Neighbors are decided using the similarities calculated in the previous step and by applying a Pareto dominance based method. In this method, drugs not dominated by other drugs are selected as neighbors. Dominance relation among drugs is decided by Eq. 4, where *d*_*i*_ and *d*_*j*_ represent drugs and *f* indicates features. According to this equation, if drug *d*_*i*_ has at least one higher similarity value than drug *d*_*j*_ and no lower similarity values than drug *d*_*j*_, then drug *d*_*i*_ dominates drug *d*_*j*_.

4 $$ dom(d_{i},d_{j}) = \left\{ \begin{array}{l l} 1.0 & \quad \forall f \; d_{i}(f)\geq d_{j}(f) \text{ and} \\ & \quad \exists f \; d_{i}(f) > d_{j}(f)\\ 0.0 & \quad \text{otherwise} \end{array} \right.  $$

**Fig. 2:**
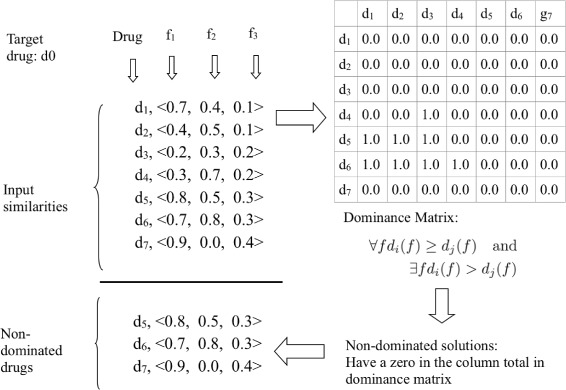
Example input and non-dominated solutions

An example input and non-dominated solutions are given in Fig. 2, where the data-set is composed of eight drugs and the target drug is identified as drug *d*_0_. Similarities between drugs for each feature *f*_*i*_ are also listed. First, based on these similarities dominance matrix is created using Eq. 4. Then non-dominated drugs (i.e., drugs with zero column total in the dominance matrix) are selected as neighbors. In this example, *d*_5_, *d*_6_ and *d*_7_ are selected as the drugs most similar to the target drug.

As explained in [29], the application of Pareto dominance based approach on a single iteration may provide less than the predefined number of neighbors. In order to collect as many neighbors as predefined, an iterative process can be applied. In each iteration, first, non-dominated neighbors are found and are removed from the first set of candidates. Then iterations are executed until the predefined number of neighbors are collected. At the end, if the collected number of neighbors is more than the predefined number (i.e., non-dominated drugs found in the last iteration are more than expected), neighbors can be pruned into exact number of neighbors or neighbors list may remain as it is. These preferences are identified in [30]; they are called Multi-Objective Optimization Type (MOT) setting which could be explained as follows.

- Only_Dominates (OD): Execute single iteration to find non-dominated neighbors. Number of non-dominated drugs is not set, and it depends directly on similarity values.
- N_Dominates (ND): Execute multiple iterations to find non-dominated neighbors. Number of non-dominated drugs is set exactly to N, i.e., pruning is applied when necessary.
- At_Least_N_Dominates (AND): Execute multiple iterations to find non-dominated neighbors. Number of non-dominated drugs is set to at least N, i.e., pruning is not applied.

#### Item selection

In this step, items to be recommended are selected. For the problem investigated in this study, selected items are diseases for which the target drug could be re-positioned. First candidates are identified by collecting items which have related neighbors, i.e., some diseases are listed as indicators for neighbor drugs. For each candidate item (disease), a score is calculated by Eq. 5, where the score is denoted *score(c,t)*, candidate item (disease) is denoted *c*, the target is denoted *t*, and the neighbor is denoted *n*. Similarity between the target and neighbor drugs is given as *sim*(*t,n*). The function *f*(*n,c*) represents neighbor drug- candidate disease relationship score given in the input data. It is possible to have this score different from zero and one, but our data-set is represented as binary vectors to indicate whether a drug has a relation with a disease or not, and the values of f(n,c) is either one or zero. Higher item selection score means the target drug has a more promising relation with the candidate disease.

5 $$ score(c, t) = \sum_{n \in \text{Nghb}} sim(t,n) \times f(n,c)  $$

For computing the score, two different settings can be used. They are called Item Selection Type (IST) settings, and they are described as follows:

- Sum (SUM): Without considering similarities between the target and neighbor drugs, votes (summation of *f*(*n,c*) values) are calculated for each candidate. Items (disease) which have highest number of votes are presented in the output list. This settings has been already described in [30].
- Weighted Sum (WSUM): For the summation, *sim*(*t,n*) value is also included, where more similar drugs have more weight in the prediction. Items (diseases) which have highest scores are included in the output list.

## Results and discussion

For the evaluation, we used the same data-set used by Zhang et al. [41], which they have shared in their website (see http://astro.temple.edu/~tua87106/drugreposition.html). In the following subsections, we explain the data-set, evaluation metrics and evaluation results.

### Data set

As the golden data-set, we used the same drug-disease data provided by Zhang et al. [41]; the dataset was also used by Li et al. [22]. The dataset integrates three data sources, namely chemical data, protein data and side-effects data.

- Chemical data contains 122,022 links between 1007 drugs and 881 PubChem [36] chemical substructures. Each drug is represented as a binary vector, where each entry indicates presence or absence of related chemical substructure. Sparsity of the data-set is about 86.25%.
- Protein data contains 3152 associations between 1007 drugs and 775 UniProt target proteins. Target drugs are generated using DrugBank [38]. Sparsity of this data is 99.60%.
- Side-effects data contains 61,102 connections between 888 drugs and 1385 side-effects. Sparsity ratio is 95.03%. Information related to this data has been generated from SIDER database [21].

Each data source contains information about a single feature, and features are represented as a binary vector. Drugs listed in each data source are not necessarily the same. Based on this, the overall data-set (combination of all three data-sources) contains more than 1007 drugs. Since drugs in each data source may be different, drugs may have missing information about one or more features.

In this work, after obtaining the dataset of Zhang et al. [41], we applied a preprocessing step to collect a list of drug names and for the mapping to drug names in chemical, protein and side-effects data sources. During this process, we noticed that some drugs may have different names (synonyms). For example, we found that one drug is referred to as *Ursodiol* in chemical data, while it is referred to as *Ursodeoxycholic acid* in both protein and side-effect data. We looked up synonyms from DrugBank website [8]. As a result of the preprocessing step, we obtained 1224 different drugs with the mappings of their names^2^.

The golden dataset, which is also provided by Zhang et al. [41], contains associations between 799 drugs and 719 diseases, with 3250 treatment relations (edges). However, not all drugs listed in this dataset are listed in the input data sources (chemical, protein and side-effect data). Since it is nearly impossible to predict targets of a drug without any prior information, we did not consider those drugs in the process. The resulting golden dataset contains 781 drugs, 719 diseases and 3179 associations^3^. Here, it is worth mentioning that this dataset may lack information on recent drug-disease relations which were not available at the time it was created by Zhang et al. [41].

**Fig. 3:**
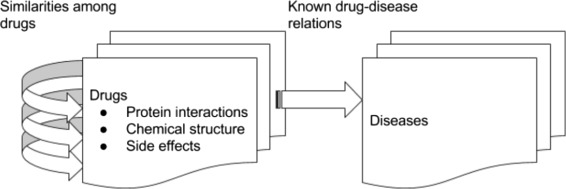
Drug-disease relations

The overall structure of the dataset is shown in Fig. 3. Drug-drug relations are created based on their similarities to each other using the above-mentioned data sources, namely, protein interactions, chemical structures and side-effects. These data sources are represented as binary matrices, where rows represent drugs and columns represent proteins, chemical compounds or side-effects, depending on the information in the data source. In the binary matrix, 1 and 0 are used to indicate whether a relationship (like causing a certain side-effect) exists or not, respectively. Drug-disease relations are also represented as binary matrix, where drugs are listed as rows and diseases are listed as columns. If a drug in a row is known to be used for the treatment of a disease in a column, the intersection cell is set to 1; otherwise the cell is set to 0. In all the data, drugs and diseases have been represented using their names as text; no other identifier has been used.

### Evaluation metrics

For the evaluation, precision@k, recall@k and F1-measure metrics are used. The formulas for computing these metrics are given in Eqs. 6, 7 and 8, where *k* indicates output list length, *tp* denotes true positives, i.e., predicted and actually indicated diseases, *fp* denotes false positives, i.e., predicted but actually not indicated diseases, and *fn* denotes false negatives, i.e., not predicted but actually indicated diseases.

6 $$ Precision_{k} = \frac{tp_{k}}{tp_{k}+fp_{k}}  $$

7 $$ Recall_{k} = \frac{tp_{k}}{tp_{k}+fn_{k}}  $$

8 $$ F1-measure = 2* \frac{Precision*Recall}{Precision+Recall}  $$

**Fig. 4:**
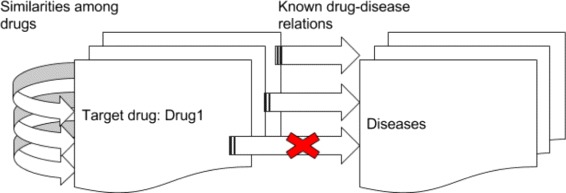
Leave-one-out strategy

For the evaluation, we used the leave-one-out strategy, i.e., we removed the target drug and its relations from the dataset and used the rest in the calculation (Fig. 4). For example, for target drug *Irbesartan* we removed drug-disease relations that already exist in the input dataset. These diseases are known to be cured by *Irbesartan*, and hence they have been used for validation. The output of our methodology, i.e., predictions of diseases which can be cured by “Irbesartan” are compared to this validation set. For each target drug, we computed the metrics explained above and we reported the average results. Also, noticing the fact that recent drug-disease relationships don’t exist in the input dataset (since those relations were not known at the time when the dataset was generated), we additionally compared our predictions to the novel clinical tests, using ClinicalTrials.gov website.

### Evaluation results

**Fig. 5:**
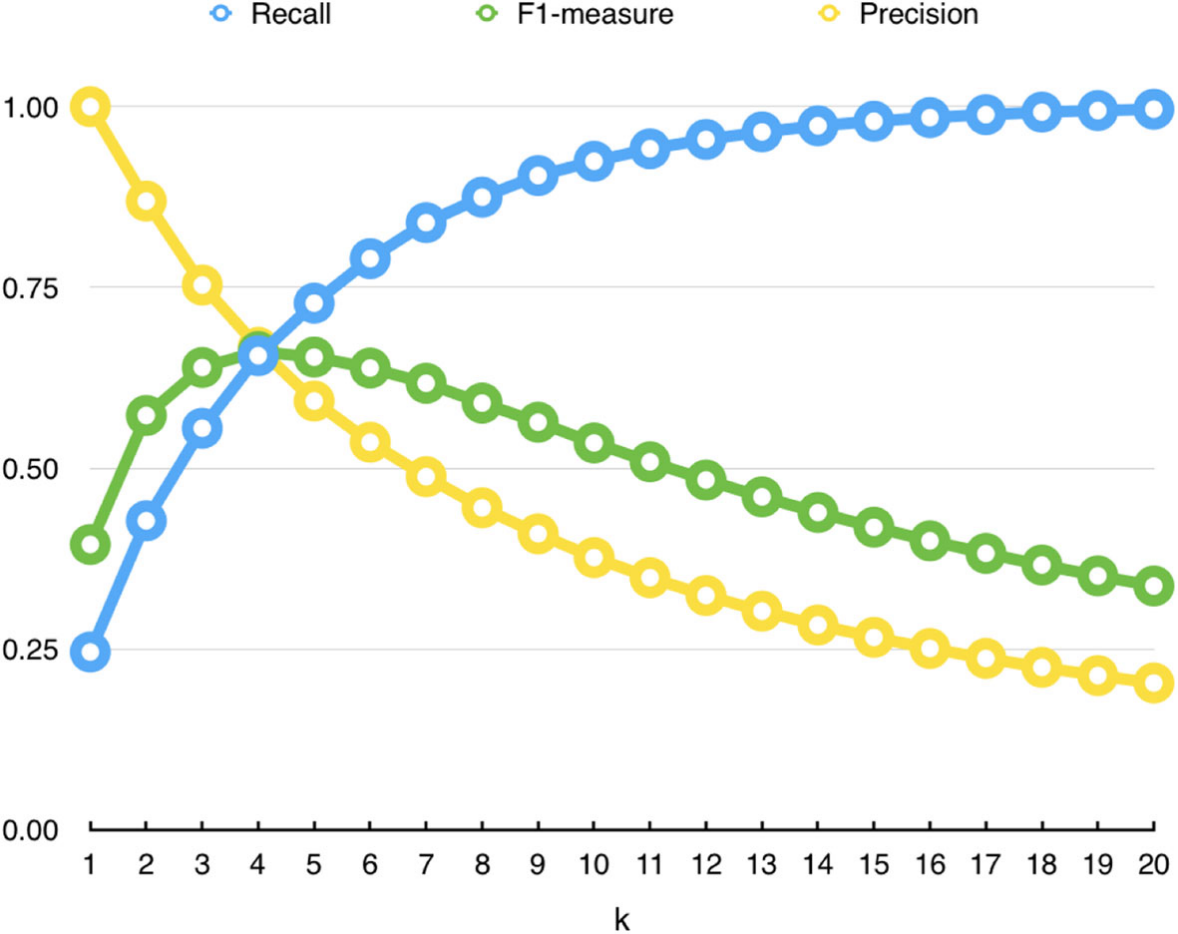
Upper bounds recall, precision and F1 measures

We first calculated upper bounds of the performance metrics. Figure 5 shows the upper bounds of precision, recall and F1-measure for different *k* values. As expected, precision is inversely proportional to the value of *k*, i.e., best precision is achieved for smaller k values, and it decreases as *k* increases. Recall has reverse behavior compared to precision, i.e., it increases as *k* increases. F1-measure, which is the harmonic mean of precision and recall, reaches its best value when *k* is equal to 4. We stopped the evaluation when *k*=20, since recall has already reached 0.9966.

Setting the output list size to exactly *k* has one drawback because not all drugs in the golden dataset have association with *k*-many diseases. If output list size is set to exactly *k*, then some predictions will always be wrong. For example, assume that *k* is set to 10, and for target drug *d*_1_, disease associations in the golden set is 5. Then, precision will be at most 0.5. However, if *k* is set to 10 in a loosely way to allow the methods to predict *at most* 10 items, precision may become 1.0. The proposed method has the ability to predict at most *k* associations and does not make any random guesses. We argue that making random guesses for drug-repositioning is not an appropriate idea. It will reduce the benefits of computational drug repositioning compared to traditional methods.

**Fig. 6:**
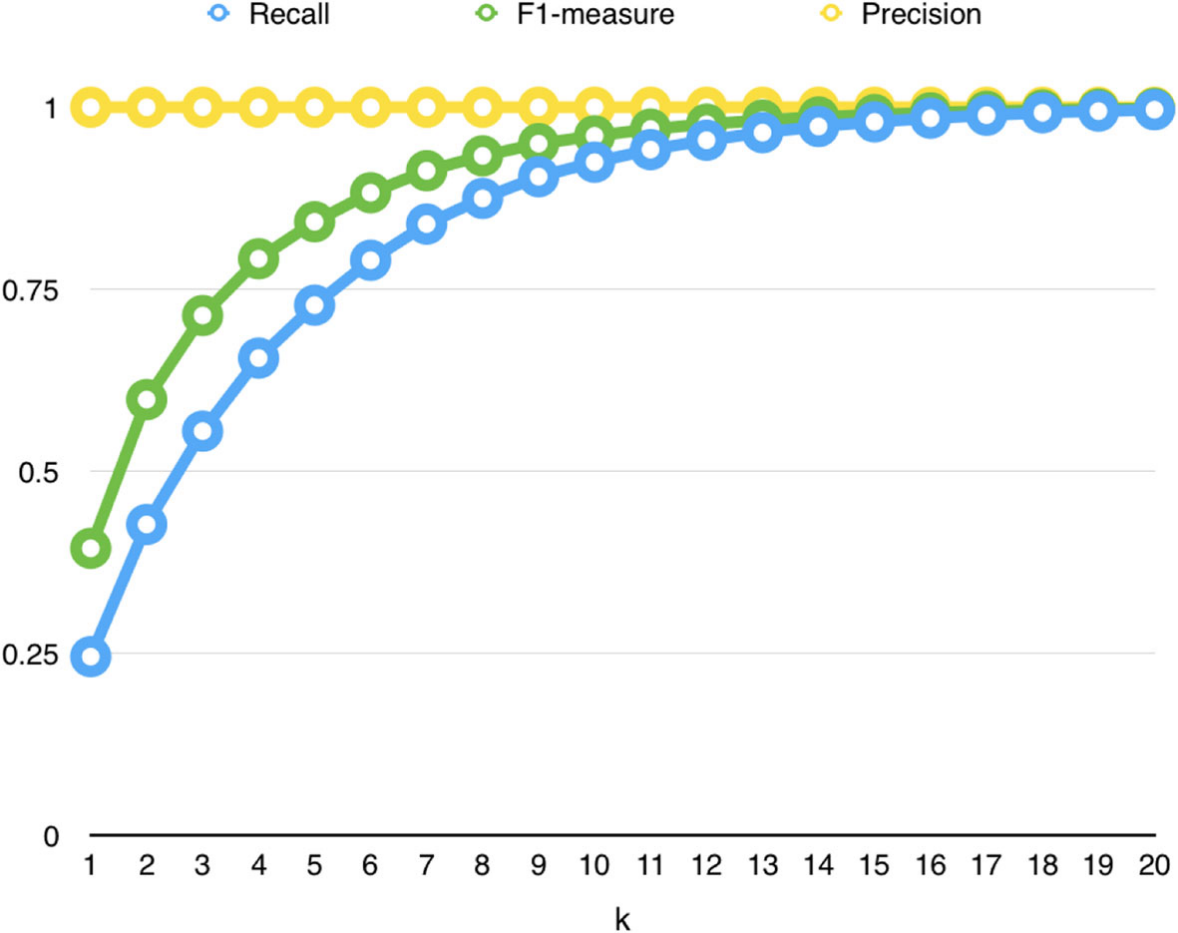
Upper bounds of recall, precision and F1 measures when random guess is not allowed

Figure 6 shows upper bounds of precision, recall and F1-measure when random guess is not allowed. In this figure, precision is always 1.0, as expected. The recall increases as *k* increases, and this leads to increase in F1-measure. In our method, we used the value of *k* in a loosely way, such that the method can’t produce more than *k* predictions. However, it is possible that the proposed method predicts less than *k* drug-disease relations per target drug. Here it is worth noting that the process of making at most *k* predictions (without guesses) is more challenging, since the method should decide on the best output list size for each target, in addition to making the best prediction.

We conducted experiments using several settings. We used different similarity metrics, namely Multi-Objective Optimization Type (MOT), and Item Selection Type (IST). For similarity type settings, we concentrated on four different settings that use Cosine similarity, Jaccard similarity or Smith-Waterman sequence alignment based similarity scores for various features, namely chemical, protein and side-effect features. In the first setting (CCC), Cosine similarity is used for all features. In the second setting (JJJ), Jaccard similarity is used for all features. In the third setting (JJC), Jaccard similarity is used for chemical and side-effect features and Cosine similarity is used for protein feature. For the last setting (JJS), Jaccard similarity is used for chemical and side-effect features and Smith-Waterman sequence alignment based similarity is used for protein feature.

**Fig. 7:**
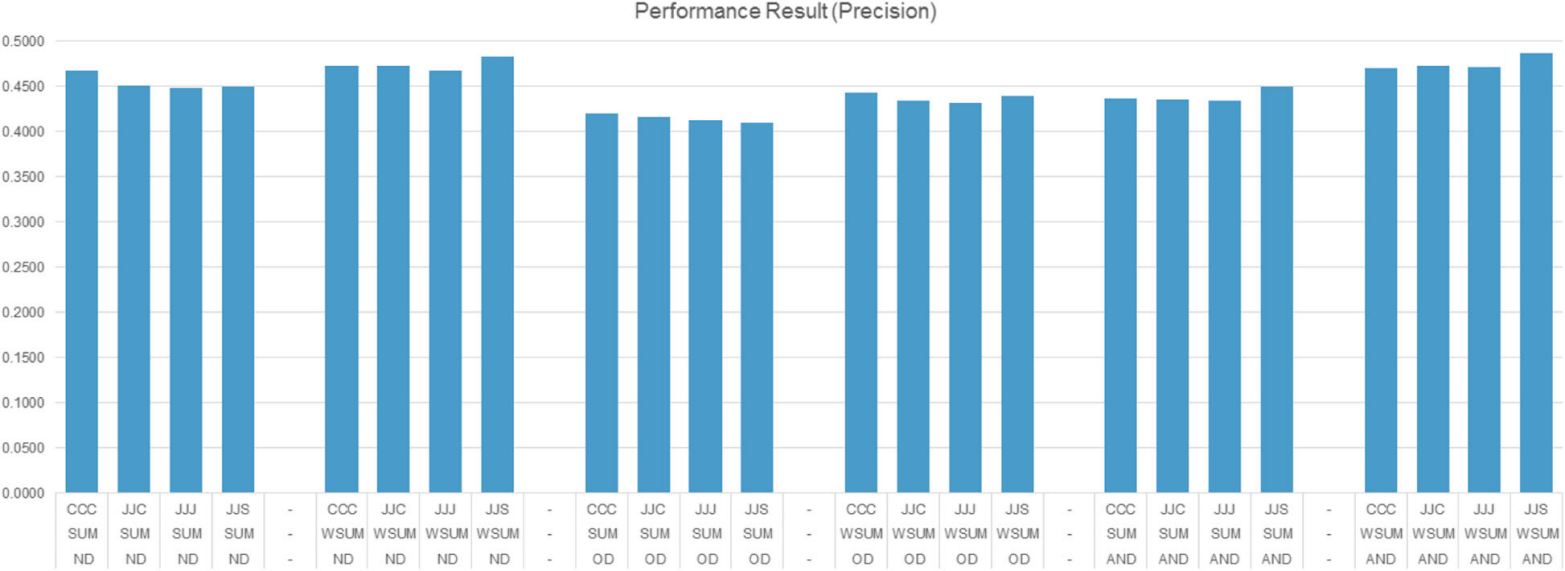
Performance results (Precision)

**Fig. 8:**
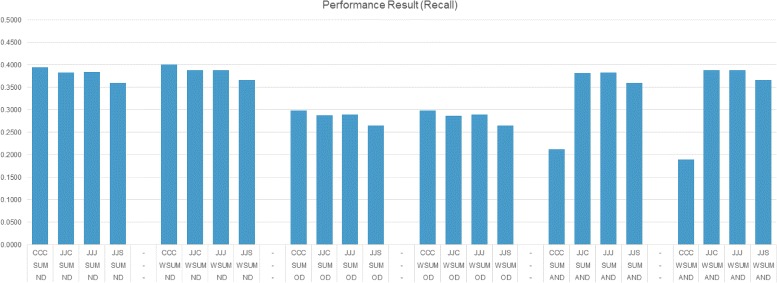
Performance results (Recall)

**Fig. 9:**
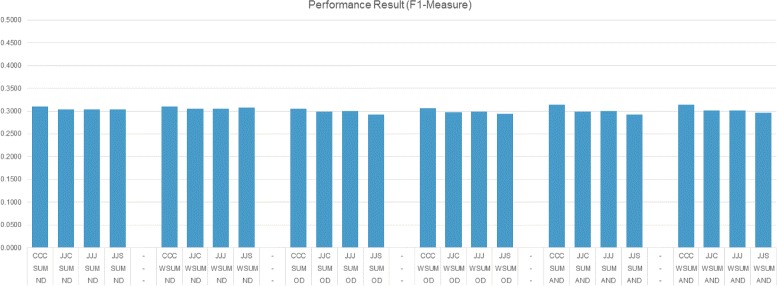
Performance results (F1-Measure)

In the experiments, we need to set two variables, namely neighbors count (*N*) and output list size (*k*). We set maximum neighbor count and output list size to 20. Instead of testing with a single value, during the experiments we set *N* and *k* to 1, 4, 8, 12, 16 or 20 and conducted experiments using the combination of *N* and *k* values. Figures 7, 8 and 9 present the best performance of the proposed method with different settings. The presented results are calculated for each *N*×*k* combinations, but only results of best performing values for the related setting are used. The settings are presented on the x-axis and each line reflects a similarity type (e.g., CCC), MOT (e.g., ND) and IST (e.g., SUM), respectively.

Figures 7 and 8 reveal that using weighted summation for item selection (WSUM) performs equally well or better than summation (SUM). ND and AND settings as MOT type perform equally well; they perform better than OD which has the limitation of choosing non-dominated neighbors on a single iteration and lead to selection of few neighbors. ND and AND have ability to choose more neighbors and performance results show that choosing more neighbors is more informative. Using different similarity measures during the calculations don’t effect the performance much. Using Smith-Waterman sequence alignment based similarity score for protein feature similarity (JJS) performs slightly better than others in terms of precision. Figure 9 shows that the performance of all settings are nearly equal. Considering all figures, observing the performance on F1-measure indicates that methods which perform good on precision do not perform good on recall, and methods which perform good on recall do not perform good on precision.

Table 1 reports best performance of the settings which use different similarity metrics in more detail. Performance results of each setting are grouped together. In each group, we report the approach which produced best precision, best recall and best F1-measure scores. As expected, precision performed better when there are fewer predictions and recall performed better when there are many predictions. While listing only one disease for a target drug produced better precision, listing many (20) diseases in prediction produced the best recall. We observed that using ND or AND method as Multi-Objective Optimization Type (MOT) performed better compared to OD. During the experiments, we observed that OD (Only dominates) type usually finds few neighbors. We further observed that having more neighbors is more useful for making better prediction. When we look at Item Selection Type (IST), we observe that using weighted sum (WSUM) performs better than using sum (SUM). This indicates that it is more informative to integrate similarity between a target drug and its neighbors. Also comparing the results in Table 1 to the upper-bounds in Fig. 6) reveals that the proposed method is able to achieve around 33% performance.

**Table 1:** The best results when different similarity metrics are used

SimType	N	k	MOT	IST	Prec.	Recall	F1
CCC-Prec.	20	1	ND	WSUM	0.4723	0.0884	0.1489
CCC-Recall	20	20	AND	WSUM	0.1894	**0.4017**	0.2575
CCC-F1	4	20	ND	WSUM	0.2636	0.3762	**0.3100**
JJJ-Prec.	12	1	AND	WSUM	0.4716	0.0862	0.1457
JJJ-Recall	20	20	ND	WSUM	0.1891	0.3888	0.2544
JJJ-F1	4	20	ND	WSUM	0.2621	0.3649	0.3051
JJC-Prec.	12	1	AND	WSUM	0.4723	0.0859	0.1453
JJC-Recall	20	20	ND	WSUM	0.1889	0.3885	0.2542
JJC-F1	4	20	ND	WSUM	0.2629	0.3652	0.3057
JJS-Prec.	12	1	AND	WSUM	**0.4864**	0.0846	0.1442
JJS-Recall	20	20	ND	WSUM	0.2036	0.3671	0.2619
JJS-F1	4	20	ND	WSUM	0.2753	0.3473	0.3071

We observed that several studies described in the drug repositioning literature prefer to present AUC-ROC (Area Under Curve - Receiver Operator Characteristic) results. However, for highly skewed data, it is stated in [6] that using precision-recall is more informative than using ROC curves. Prediction based on data which has fewer positive relations and many negative relations is commonly considered in the information retrieval literature as “searching for a needle in haystack’. The golden data we used has similar characteristics, since there are only 3179 positive relations and 558,360 negative relations. Based on this observation, we also included AUC-PR scores while presenting the performance of the proposed method and settings.

Table 2 reports the calculated AUC-PR scores of the proposed method and settings. To compute AUC-PR values of the proposed methods we used code from https://github.com/andybega/auc-pr/blob/master/auc-pr.r. The results show that using Jaccard and Smith-Waterman sequence alignment based similarity scores can lead to better performance compared to other methods, especially when the output list size is limited to few predictions (e.g., *k*=1).

**Table 2:** AUC-PR results when different similarity metrics are used

SimType	N	k	MOT	IST	AUC-PR
CCC-Prec.	20	1	ND	WSUM	0.2178
CCC-Recall	20	20	AND	WSUM	0.0584
CCC-F1	4	20	ND	WSUM	0.0839
JJJ-Prec.	12	1	AND	WSUM	0.2181
JJJ-Recall	20	20	ND	WSUM	0.0595
JJJ-F1	4	20	ND	WSUM	0.0850
JJC-Prec.	12	1	AND	WSUM	0.2184
JJC-Recall	20	20	ND	WSUM	0.0595
JJC-F1	4	20	ND	WSUM	0.0852
JJS-Prec.	12	1	AND	WSUM	**0.2252**
JJS-Recall	20	20	ND	WSUM	0.0662
JJS-F1	4	20	ND	WSUM	0.0917

We also compared our proposed method to the methods described in the literature; the results are reported in Table 3. Actually, we compared our method to the state of the art methods which were evaluated using the same dataset we used in this study, namely Li and Lu [22], Chiang and Butte [3], and Zhang et al. [41]. For the proposed method, we presented two settings which produce best precision and best recall. In the table, we have included precision, recall and F1-measure results. We have not included AUC-PR results since the methods described in the literature usually use ROC and AUC-ROC results. To be able to compare results from the proposed methods to results from other methods described in the literature, we have decided to include in the table sensitivity (recall), specificity and AUC-ROC measures as well. The importance of using AUC-PR in scale-free networks, like biological networks, is also underlined in the works conducted by Wu et al. [39] and Lotfi et al. [25]. They stated that PR curves are more informative when the distribution of relations are skewed.

**Table 3:** Comparison of the proposed method to other state of the art methods from the literature

Type	Prec.	Recall	F1	SPC	AUC-ROC
Li and Lu [22]	-	**0.7700**	-	0.9200	0.8880
Chiang and Butte [3]	-	0.7400	-	0.8500	-
Zhang et al. [41]	0.3452	0.6505	**0.4510**	-	**0.8949**
Proposed Method - JJS	**0.4864**	0.0846	0.1442	**0.9995**	0.5421
Proposed Method - CCC	0.1894	0.4017	0.2575	0.9902	0.6960

Sensitivity (recall) and specificity are used to create ROC. Equation 9 shows how specificity (SPC) is calculated. In the equation *tn* refers to true negatives, i.e, not predicted and actually not indicated diseases, and *fp* represents false positives, i.e., predicted but actually not indicated diseases. Specificity (SPC) measures performance of the methods on negative links (i.e., no indication for a disease). Finally, AUC-ROC values of the proposed method have been derived using ROCR library in R.

9 $$ SPC = \frac{tn}{tn+fp}  $$

The results reported in Table 3 show that the proposed method with JJS setting performs better than other methods in terms of precision and specificity. This indicates that this method is able to make true predictions for positive and negative relations; i.e,. its *tp* and *tn* values are high. However, it has low recall, indicating that it cannot predict all true drug-disease relations. This result is expected, since in this setting number of predictions is set to 1 (*k*=1). Actually, the upper-bound of recall when *k*=1 is around 0.25 (Fig. 6) and the proposed method is able to achieve 33% of recall performance. Other methods have lower precision and higher recall and AUC-ROC values. This reflects that those methods were able to predict many drug-disease relations (i.e., *k* has higher value in their settings), but they also listed many false relations.

The golden data we use is very skewed and has 99.44% sparsity; i.e., there are many diseases that are irrelevant to a target drug. We would argue that precision is more important than recall for this dataset and for the drug repositioning problem in general, i.e., making the right prediction for drug-disease relations is more important than finding all relations. Comparing our method to other state of the art methods from the literature shows that the proposed method can achieve higher precision, e.g., when it predicts a drug-disease relation, nearly half of those predictions are true.

Lastly, we compared our predictions to novel clinical tests, using ClinicalTrials.gov website, which collects and presents information on publicly and privately supported clinical studies of human participants around the world. From the website, we looked up a drug and disease relations predicted by the proposed method with highest precision value, i.e., Proposed Method - JJS and output list size (*k*) is 1. Comparing predictions to golden dataset reveals that the proposed method predicted 269 true positives (predicted and actually true relation) and 284 false positives (predicted, but not actually true relation). When we use ClinicalTrials.gov for comparison to novel clinical tests, we realized that 98 of the false positives, nearly 1/3 of the false positives, were actually clinically tested after the golden dataset was produced. This indicates that these predictions are actually true. For example, the relation between drug *Amifostine* and disease *Xerostomia* does not exist in the golden dataset. However, our proposed method is able to predict this relation. ClinicalTrials.gov website revealed that there is actually a relation between drug *Amifostine* and disease *Xerostomia*. In Table 4, we present an example set of predictions made by the combination Proposed Method - JJS with output list size (*k*) set to 1, together with whether these predictions are actually clinically tested or not^4^.

**Table 4:** An example set of predictions (Proposed Method - JJS and k=1)

Drug	Predicted disease	Clinical test
Amifostine	Xerostomia	TRUE
Amprenavir	Corneal Ulcer	FALSE
Arformoterol	Hypertension	TRUE
Bimatoprost	Asthma	FALSE
Buclizine	Urticaria	FALSE
Clofazimine	Vertigo	FALSE
Dexamethasone	Inflammation	TRUE
Fenoldopam	Parkinson Disease	FALSE
Irbesartan	Heart Failure	TRUE
Levodopa	Asthma	TRUE
Mazindol	Depressive Disorder	FALSE
Mephobarbital	Epilepsy	FALSE
Nitrofurantoin	Diarrhea	TRUE
Oxymetazoline	Hypotension	TRUE
Oxytetracycline	Inappropriate ADH Syndrome	FALSE
Pemirolast	Motion Sickness	FALSE
Procarbazine	Osteoarthritis	FALSE
Temozolomide	Hypertension	TRUE
Yohimbine	Postpartum Hemorrhage	FALSE
Zolpidem	Heart Failure	TRUE

## Conclusions

Drug repositioning is an essential process for linking emerging diseases to existing known and well tested drugs as opposed to seeking the development of new drugs for such diseases. The latter process is associated with several risks and costs which may not be easily affordable. Thus, repositioning has received considerable attention in industry and academia. In this paper, we described a new approach for drug repositioning which performs well compared to state of the art other approaches described in the literature. The originality of our approach is realizing the whole drug repositioning process as a recommendation process where drugs are recommended based on similarity and overlap between symptoms of diseases and effectiveness of drugs. This approach opens a new dimension in the drug repositioning literature by demonstrating how it is possible to reposition existing computation techniques developed to handle a specific domain and map them to become effective solutions for other emerging domains. We illustrated how various computing techniques may contribute to ongoing efforts for drug repositioning, and hence may help in reducing associated risks, cost and time required to identify new drugs.

One attraction of our approach is the set of features used in the process. The approaches described in the literature employ a variety of computational methods and various features of drugs and diseases to identify drug-disease coupling. The most common features used in the literature are chemical structure of drugs, protein targets interaction networks, side-effects of drugs, gene expressions and textual features. Computational drug repositioning methods use a single feature or combine multiple of them. On the other hand, our recommendation system based method is able to integrate multiple data-sources and multiple features. The method is based on Pareto dominance and collaborative filtering to identify drugs most similar to a target drug, and neighbor drugs are then used to predict new indication of the target drug. Also, we applied and compared the performance of several different settings that affect the computation. Experimental results show that the proposed method is able to achieve high precision, such that nearly half of the predictions are true. Comparison to the other methods described in the literature show that the proposed method is better at making concentrated predictions with higher true positive ratio. Having concentrated (fewer and to-the-target) predictions helps researchers in biology and chemistry who will use the output drug-disease relation predictions in their laboratory experiments. In general, the results show that it is highly promising to use a recommendation method to tack drug repositioning. In order to further our research, we intend to use a more up-to-date drug-disease relations dataset and apply the proposed method on this new dataset. We plan to use a recent database which integrates multiple data sources and presents more recent drug-disease relations [4]. We also want to integrate other known recommendation methods in handling the drug repositioning problem and to apply these methods on other (larger) datasets to observe and analyze their performance in depth. Lastly, we are aware of the fact that drug-disease relations can be organized in different ways rather than a flat structure. For example, diseases may have hierarchical relations or drugs’ features (e.g., drug-protein relations) may have multiple levels. Future studies should examine the effects of different structural representations of drug-disease relations. Another idea that future studies may focus on is the representation of drugs and diseases in the input dataset, where identifiers may be preferred to using names.

## Abbreviations

AND: At_Least_N_Dominates
ND: N_Dominates
OD: Only_Dominates ROC: Receiver operator characteristic
SUM: Sum
WSUM: Weighted sum

## Footnotes

1 We used Uniprot to collect protein sequence information and ClustalX2 for protein sequence alignment.

2 We plan to share the mappings of names on our website.

3 We will share the golden set on our website.

4 We will present on our website all predictions made by all combinations of the proposed method and similarity metrics with output list size (*k*) values.
