# Correction to: Realizing drug repositioning by adapting a recommendation system to handle the process

**DOI:** 10.1186/s12859-018-2258-3

**Published:** 2018-07-02

**Authors:** Makbule Gulcin Ozsoy, Tansel Özyer, Faruk Polat, Reda Alhajj

**Affiliations:** 1 Department of Computer Engineering, Middle East Technical University, Ankara, Turkey; 2 Department of Computer Engineering, TOBB University, Ankara, Turkey; 3 Department of Computer Science, University of Calgary, Calgary, AB Canada

## Correction

Following publication of the original article [1], the authors reported that there was an error in the spelling of the name of one of the authors.

Originally the author’s name was spelled as:

Makbule Guclin Ozsoy

The correct spelling is:

Makbule Gulcin Ozsoy
